# Molecular epidemiology of hepatitis B virus isolated from Bangladesh

**DOI:** 10.1186/s40064-016-3174-5

**Published:** 2016-09-08

**Authors:** Modhusudon Shaha, Sheikh Ariful Hoque, Sabita Rezwana Rahman

**Affiliations:** 1 Microbial Biotechnology Division, National Institute of Biotechnology, Savar, Dhaka, 1349 Bangladesh; 2 Department of Microbiology, University of Dhaka, Dhaka, 1000 Bangladesh; 3 Cell and Tissue Culture Laboratory, Center for Advance Research in Sciences, University of Dhaka, Dhaka, 1000 Bangladesh

**Keywords:** Hepatitis B virus, Genotypes, Mutations, Partial S gene, Bangladesh

## Abstract

**Background:**

Hepatitis B virus (HBV) is highly contagious and causes liver diseases. Globally more than 350 million people are chronically infected and among them above 80 % are from developing countries like Bangladesh. Resistance to existing drugs and vaccines are common phenomenon due to mutations in HBsAg ‘a’ determinant. Due to lack of data about mutations and subtypes of HBV in Bangladesh, this study strongly demands to be documented. Here, we determined the genotypes and subtypes of HBV prevalent in Bangladesh, and their genomic mutations associated with vaccine and drug resistance.

**Results:**

Among 385 samples, a total of 54 (14 %) were found HBV positive, of which 19 samples were subjected to be sequenced. After bioinformatic analysis, we found Genotype D as predominant genotype (73.7 %) with subtypes ayw_3_ (64.3 %) and ayw_2_ (35.7 %), followed by genotype A with subtype adw_2_ (15.8 %), and then genotype C with subtype adr (10.5 %). A significant number of mutations (Thr118Val, Thr125Met, Thr126Ile, Pro127Thr, Ala128Val, Thr131Asn/Ser, Thr/Ser143Leu/Met) were found in ‘a’ determinant region which may admit resistance to the available vaccines and failure of HBsAg detection.

**Conclusions:**

This comprehensive study have clinical importance like disease diagnosis and treatment. It emphasizes HBV infected patients to do molecular diagnosis for choice of anti-viral drugs and effectiveness of vaccines for proper treatment.

## Background

Hepatitis B virus (HBV) infects over 350 million people globally per year and causes serious acute and chronic liver diseases (Mast et al. 2005; Norder et al. 1993, 1994). Globally, more than 300 million people have chronic HBV infection and approximately two billion are infected (Scheiblauer et al. 2010; Shaha et al. 2015). HBV integrates its genome into liver cell and can reside there for long time which is seem to develop HBV carrier state and thereby, may cause damage to the liver permanently (Suppiah et al. 2014). In Bangladesh, HBV is one of the major causes of hepatocellular carcinoma (HCC), which is known to be a leading cause of death in the world (Suppiah et al. 2014).

Genomic variation of HBV genotypes is determined by genomic relatedness, where the standard cut-off of divergence is about 8 % (Bowyer and Sim 2000). In Bangladesh, the most prevalent genotype is genotype C among the chronic infection of HBV in one study (Rahman et al. 2016) and genotype D among the patients with chronic infection in a tertiary-care hospital in another study (Mamun Al et al. 2006); however the data on subtypes and mutations in surface (S) gene that contains ‘a’ determinant region are not available. The HBsAg ‘a’ determinant region is located between amino acids 124–147 within the major hydrophilic region (MHR amino acids 99–169) of this S gene encoded protein (Velu et al. 2008). Genotyping and Subtyping of HBV is totally based on increasing of variety of antibodies to the ‘a’ determinant (Moradi et al. 2012). Mutations in this region causes vaccine escape as well as HBsAg detection failure mutants through alteration of antigenicity of the protein (Carman et al. 1990).

On the other hand, drug resistance can be caused due to high mutation rate of polymerase gene, which made HBV infection unpreventable even after vaccination. These mutations can be raised mostly among chronic patients who take anti-viral drugs regularly. Vaccine and anti-viral drug resistance have been described as significant factors to treatment failure of hepatitis B as stated by Locarnini and Mason (Locarnini and Mason 2006).

Sequencing part of surface gene from 370 to 861 nt (491 bp) can be termed as partial S gene sequencing that can reveal genotyping, subtyping and common mutations as efficiently as total S gene sequencing (Wang et al. 2013). This small part of the S gene is conserved and contains ‘a’ determinant region as well as many drug resistant and vaccine mutant sites (Wang et al. 2013). Recent reports in different areas of the world, showing the anti-viral drugs resistant strains of HBV, strongly alert the need for monitoring drug resistant and vaccine mutant sites along with mutations in other genes of the genome of HBV (Sayan et al. 2010; Pastor et al. 2009; Han et al. 2009). Herein, we analyzed the partial S gene to determine the genotypes, subtypes and possible mutations in partial S gene of HBV genome through polymerase chain reaction (PCR) and sequencing.

## Methods

### Sample selection

A total of 385 patients with jaundice like illness were included in this study and blood samples were collected from different medical college hospitals of Dhaka city. This study was ethically cleared by ethical committee of Dhaka Medical College, Dhaka-1000, Bangladesh (reference number: DMC-MEU/ECC/2014/16). The samples were collected after obtaining a written consent from every patient. None of the patients were HBV vaccinated. We selected the patients with jaundice like illness who might have been infected by hepatitis B viruses because jaundice is one of the main symptoms of hepatitis B infection.

### Serological analysis

Serum samples were allowed to be clotted for approximately 10 min and centrifuged at 5000 rpm for 5 min to separate serum from blood. The obtained serum samples were subjected to analyze Hepatitis B surface antigen (HBsAg) by Enzyme Linked Immune Sorbent Assay (ELISA) using ELISA kits (JAJ International Inc., CA, USA).

### DNA extraction

HBV-DNA of several randomly selected HBsAg positive samples was extracted from 200 µl of plasma using pathogen kit (Stratec molecular, Berlin, Germany) according to manufacturer’s instruction. Extracted DNA was stored at −20 °C until use.

### Polymerase chain reaction and DNA amplification

Partial S gene was targeted to be amplified in this study. A product of 491 base pairs of partial S gene was amplified using 5′-TCGCTGGATGTGTCTGCGGCGTTTTAT-3′ and 5′-ACCCCATCTTTTTGTTTTGTTAGG-3′ primer pair as forward and reverse primers respectively corresponding to nucleotide position 370–861 (Wang et al. 2013). Four microliter (µl) of extracted DNA was added into the mixture containing 12.5 µl of 2× MiFi Mix, 1.0 µl of each primer (10 pmol) and 6.5 µl nuclease free water making total volume of 25 µl reaction. Then the mixture was amplified using a PCR protocol as follows: 12 min at 95 °C, 35 cycles of 1 min at 94 °C, 1 min at 52 °C, 1 min at 72 °C, and a final elongation step of 7 min at 72 °C. The amplification products of 491 bp were analyzed by agarose gel electrophoresis (AGE) on 1.5 % agarose gel stained with ethidium bromide to observe HBV-DNA positive and negative samples, and viewed under UV illumination.

### Post PCR purification and sequence determination

The corresponding amplicons were purified using the PCR clean-up system (Promega Corporation, Madison, WI, USA) according to the manufacturer’s instruction. Final elution contained 50 µl of purified PCR products from which 10 µl was reanalyzed on 1.5 % agarose gel to make sure that the purification step was performed precisely. Sequencing was done with the same primers used to amplify the 491 bp partial S gene sequences.

### Determination of genotypes and subtypes

After obtaining the sequences of partial S gene, NCBI-BLAST (BLASTn) was performed to get the sequence similarity with other sequences deposited in the NCBI site. The sequences were also analyzed using EMBOSS Transeq tool of EMBL-EBI server (McWilliam et al. 2013) to find all six frames of in silico translation of proteins and subjected to protein BLAST (PSI) against NCBI protein database to find the correct open reading frame (ORF) of the translated protein. Experimental sequences were aligned using MEGA version 5 tools (Tamura et al. 2011) and genotypes were determined by constructing phylogenetic tree. Phylogenetic tree was constructed by Maximum Likelihood Statistical Method with bootstrap replication of 100 times and analysed using Kimura 2-parameter model with uniform rates among sites. The obtained sequences were aligned by Clustal Omega method (Larkin et al. 2007). The reference sequences for this alignment were local and foreign S gene sequences collected from NCBI GeneBank.

### Analysis of mutations

For analyzing respective mutations that are associated with resistance to vaccine and anti-viral drugs the sequences were aligned with standard hepatitis B sequence (Accession number: AB033559) from GenBank database (Moradi et al. 2012) by MEGA version 5 software (Tamura et al. 2011) and visualized using Jalview software (Waterhouse et al. 2009). Amino acid codons 122, 160, 127, 159 and 140 of S gene were used to predict HBsAg subtypes as described elsewhere (Purdy et al. 2007).

### Sequence deposition

Partial CDS of S gene sequences is deposited in GenBank under accession numbers: KP240636–KP240654.

## Results

### Genotype D as most prevalent genotype in Bangladesh

**Fig. 1:**
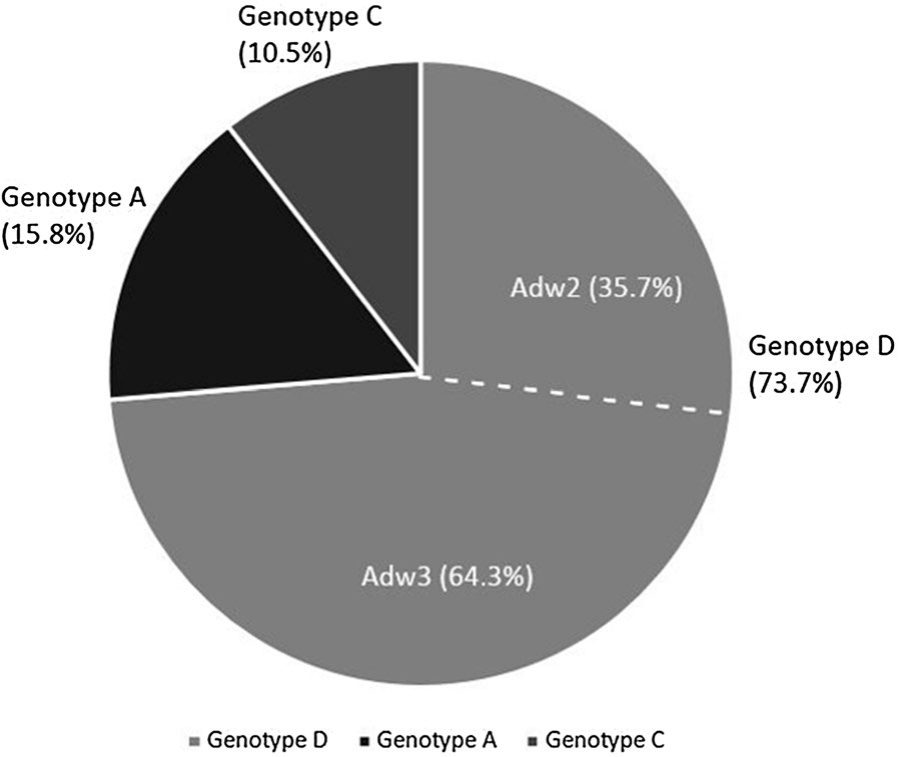
Distribution of HBV genotypes in Bangladesh

A total of 54 (14 %) samples were observed HBsAg positive. Among them 19 samples were randomly selected to be analyzed by PCR and then nucleotide sequencing. After phylogenetic analysis, 14 (73.7 %) samples were found as genotype D, followed by genotype A (15.8 %) and then genotype C (10.5 %). Phylogenetic tree revealed the similarity of these sequences varying regionally across the world. Furthermore, genotype D was comprised of two subtypes namely, ayw_3_ (64.3 %) and ayw_2_ (35.7 %), whereas, subtype adw_2_ and adr were specific for genotype A and genotype C respectively as shown in Fig. 1.

### Evolutionary relationship of our HBV with other circulating HBVs

**Fig. 2:**
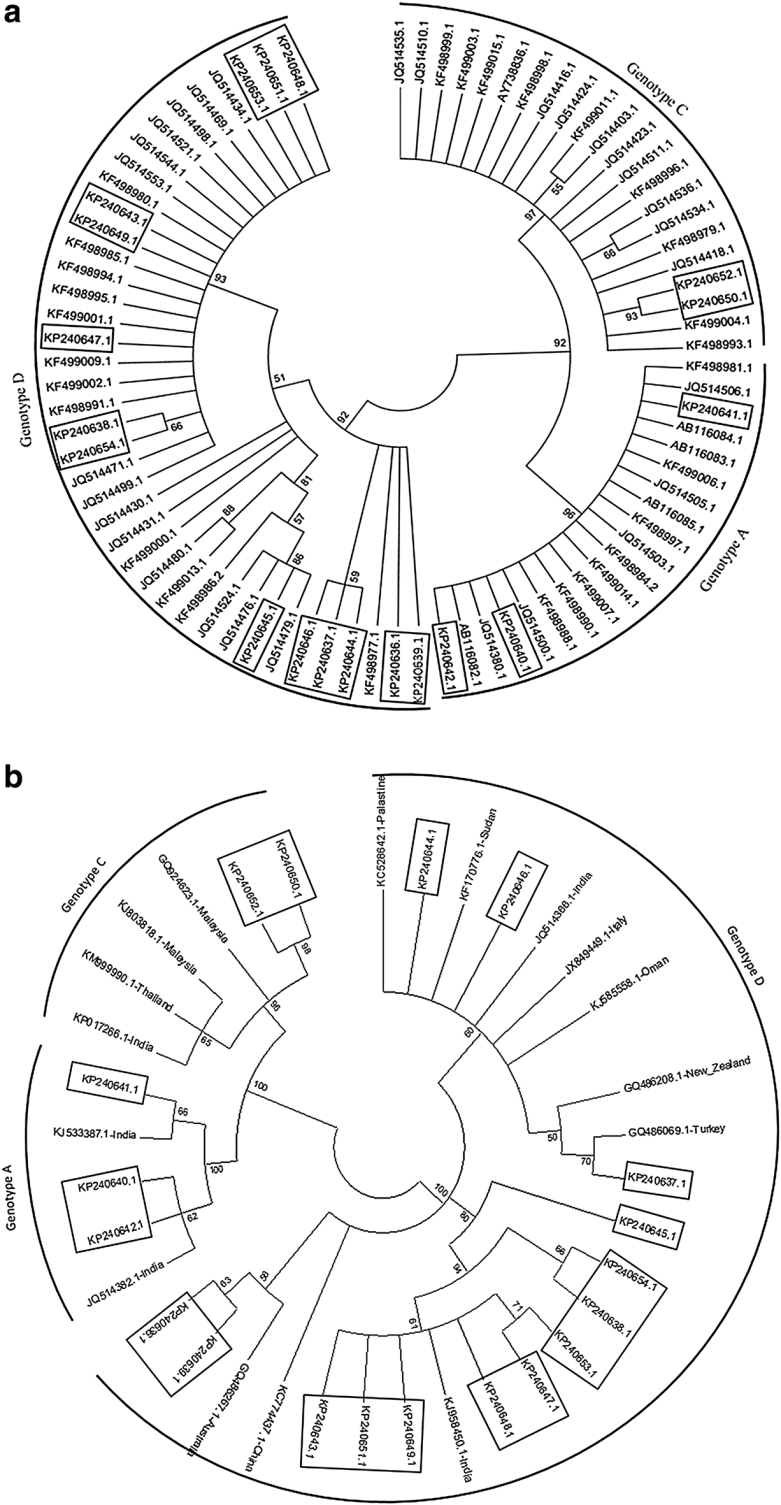
Phylogenetic analysis of HBV sequences with **a** different existing Bangladeshi strains and **b** foreign strains collected from NCBI GenBank. Our experimental sequences have been marked with *boxes*

**Fig. 3:**
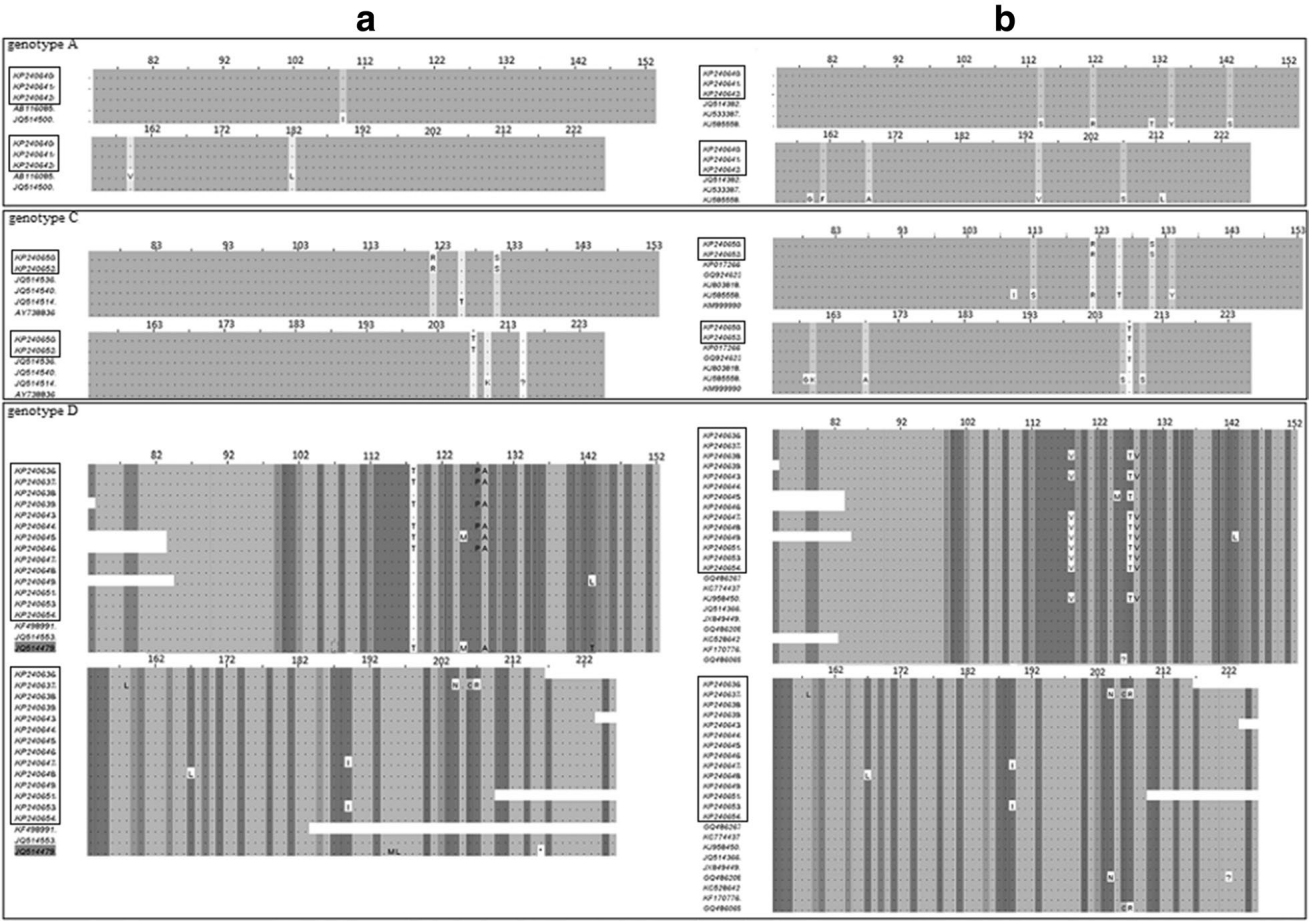
Alignment of HBV sequences with **a** different Bangladeshi strains and **b** foreign strains. Our experimental sequences have been marked with *boxes*. Matched amino acids were presented as *dot* and mismatched were shown in *symbols*. Alignment was done by MEGA v5.0 software and visualized using Jalview v2.8.1 software

After constructing phylogenetic tree, we observed strong similarity of our partial S protein sequence with other Bangladeshi sequences (up to 97 %) and sequences from India (up to 100 %) and Malaysia (96 %) as shown in Fig. 2. However, sequences comprising genotype D were evolutionarily diverted (Fig. 2). On the other hand, there were several amino acid mutations like K122R (amino acid Lysine at 122 position of S protein instead of Arginine), T131S and I208T in genotype C (Fig. 3). The above mutations might have possible effect on structural and functional activity of S protein which might be a significant cause of resistance. Phylogenetic tree of above alignment revealed that our sequences were closely related with other Bangladeshi existing sequences with a similarity of more than 90 % (Fig. 2a). Phylogenetic analysis revealed the highest similarity of genotype A, C and D sequences with the sequences from India, Malaysia and India respectively (Fig. 2b).

### Multiple mutations of our sequences in ‘a’ determinant region

Several mutations were observed in ‘a’ determinant region of S gene such as, T118V, T125M, T126I, P127T, A128V and T/S143L/M according to different genotypes of HBV (Table 1). These mutations probably have strong impact on functions of S gene especially resistance to different drugs as well as vaccine.

**Table 1:** Possible mutations in ‘a’ determinant region in the current sequences of this study

Observed genotypes	Sequences	Possible mutations
A	KP240640	No mutations
	KP240641	No mutations
	KP240642	No mutations
C	KP240650	Thr126Ile
	KP240652	Thr126Ile
D	KP240636	No mutations
	KP240637	No mutations
	KP240638	Thr118Val, Pro127Thr, Ala128Val
	KP240639	No mutations
	KP240643	Thr118Val, Pro127Thr, Ala128Val
	KP240644	No mutations
	KP240645	Thr125Met, Pro127Thr
	KP240646	No mutations
	KP240647	Thr118Val, Pro127Thr, Ala128Val
	KP240648	Thr118Val, Pro127Thr, Ala128Val
	KP240649	Thr118Val, Pro127Thr, Ala128Val, Thr/Ser143Leu/Met
	KP240651	Thr118Val, Pro127Thr, Ala128Val
	KP240653	Thr118Val, Pro127Thr, Ala128Val
	KP240654	Thr118Val, Pro127Thr, Ala128Val

## Discussion

As the severity of HBV is increasing day by day and resistance to the anti-viral drugs and vaccines is raising exponentially, it is essential to demonstrate the mutation status of HBV genome across the world. Hence, the purpose of this study was to determine the genotypes and subtypes of HBV prevailing in Bangladesh and to demonstrate possible mutations in ‘a’ determinant region that might be responsible for resistance to different anti-viral drugs and vaccines.

In this study, we found genotype D (73.7 %) as most prevalent in Bangladesh, followed by genotype A (15.8 %) and then genotype C (10.5 %), which is consistent with other study done in India (Ismail et al. 2012). We used different existing Bangladeshi and foreign sequences of different genotypes as well as a standard GenBank sequence (Accession number: AB033559) (Moradi et al. 2012) from NCBI GenBank to determine the genotypes in Bangladesh and aligned the sequences of those genotypes to observe subtypes. In Bangladesh, the predominant subtypes associated with genotype D are ayw_3_ (64.3 %) and ayw_2_ (35.7 %), and subtypes adw_2_ and adr were specific for genotype A and C respectively. One previous study in Bangladesh documented the predominant genotype in Bangladesh is genotype D which was in accordance with our study (Mahtab et al. 2008; Mamun Al et al. 2006).

We demonstrated several amino acid variations in genotypes A, C and D comparing with the previously documented NCBI sequences. Whereas, genotype A was more conserved than genotypes C and D as shown in Fig. 3. Genotype D was found more susceptible to surface gene mutations (Table 1).

The highest similarity of genotype A and D was found with the sequences from India. However, genotype C was found most similar with the sequences from Malaysia, which are highly communicated countries by Bangladesh.

The genomic mutation in partial S region of HBV have clinical importance. Mutations in ‘a’ determinant region are associated with functional properties of HBV as described above. Different mutations in ‘a’ determinant were found in our sequences as shown in Table 1. Among which, Thr118Val, Thr125Met, Pro127Thr, Ala128Val and Thr/Ser143Leu/Met were observed in genotype D sequences, which is thought to have strong impacts on functions of S gene. For example, substitution Pro127Thr has been alarmed as great public health significance. Patients with this mutation lack to exhibit quantifiable HBsAg as declared in different studies (Simon et al. 2013; Scheiblauer et al. 2010). Furthermore, Sequences of genotype C were found to have Thr126Ile and Thr131Ser mutations, and genotype A sequences have only Thr131Asn mutation in ‘a’ determinant region. Although activity of these mutations was not documented, they might lead to the conformational change of ‘a’ determinant region and as a result, vaccine would be less immunogenic to induce an antibody response even after complete vaccination. Hence, this study recommend to demonstrate experimentally whether these mutations have any resistant property to anti-viral drug or vaccine.

## Conclusions

This study is unique for three reasons (1) a detail mutation profile of ‘a’ determinant region of S gene was delineated (2) amino acid comparison of our sequences with different existing Bangladeshi and foreign sequences was described and lastly (3) this is the first comprehensive study detecting the mutations and subtypes of HBV in Bangladesh. This study has the importance to direct clinicians to diagnose the disease properly and decide the choice of drugs or vaccines for the treatment of hepatitis B and HBV carriers.

## Abbreviations

AGE: agarose gel electrophoresis
bp: basepair
ELISA: Enzyme Linked Immune Sorbent Assay
HBV: hepatitis B virus
HBsAg: hepatitis B surface antigen
HCC: hepatocellular carcinoma
MHR: major hydrophilic region
nt: nucleotide
ORF: open reading frame
